# Metaproteomics to Decipher CF Host-Microbiota Interactions: Overview, Challenges and Future Perspectives

**DOI:** 10.3390/genes12060892

**Published:** 2021-06-09

**Authors:** Pauline Hardouin, Raphael Chiron, Hélène Marchandin, Jean Armengaud, Lucia Grenga

**Affiliations:** 1 Laboratoire Innovations technologiques pour la Détection et le Diagnostic (Li2D), Université de Montpellier, 30207 Bagnols-sur-Cèze, France; pauline.hardouin@cea.fr; 2 Département Médicaments et Technologies pour la Santé (DMTS), Université Paris-Saclay, CEA, INRAE, SPI, 30200 Bagnols-sur-Cèze, France; jean.armengaud@cea.fr; 3 HydroSciences Montpellier, CNRS, IRD, Université de Montpellier, Centre de Ressources et de Compétences de la Mucoviscidose, CHU de Montpellier, 34093 Montpellier, France; r-chiron@chu-montpellier.fr; 4 HydroSciences Montpellier, CNRS, IRD, Université de Montpellier, Service de Microbiologie et Hygiène Hospitalière, CHU Nîmes, 34093 Nîmes, France; helene.marchandin@umontpellier.fr

**Keywords:** microbiota, cystic fibrosis, proteomics, mass spectrometry

## Abstract

Cystic fibrosis (CF) is a hereditary disease caused by mutations in the CF transmembrane conductance regulator (*CFTR*) gene, triggering dysfunction of the anion channel in several organs including the lung and gut. The main cause of morbidity and mortality is chronic infection. The microbiota is now included among the additional factors that could contribute to the exacerbation of patient symptoms, to treatment outcome, and more generally to the phenotypic variability observed in CF patients. In recent years, various omics tools have started to shed new light on microbial communities associated with CF and host–microbiota interactions. In this context, proteomics targets the key effectors of the responses from organisms, and thus their phenotypes. Recent advances are promising in terms of gaining insights into the CF microbiota and its relation with the host. This review provides an overview of the contributions made by proteomics and metaproteomics to our knowledge of the complex host–microbiota partnership in CF. Considering the strengths and weaknesses of proteomics-based approaches in profiling the microbiota in the context of other diseases, we illustrate their potential and discuss possible strategies to overcome their limitations in monitoring both the respiratory and intestinal microbiota in sample from patients with CF.

## 1. Introduction

The genetic mechanism underlying cystic fibrosis (CF) is linked to a combination of genetic mutations in the gene coding for the cystic fibrosis transmembrane conductance regulator (CFTR). Over 2000 known *CFTR* variants are now associated with a wide range of biological and functional consequences [1] resulting in poor hydration of mucus and impaired function in respiratory, digestive, and reproductive organs. Although recent advances in the molecular understanding of the functional impact of specific *CFTR* variants have revolutionised drug development, phenotypic differences between CF patients expressing the same genotypic *CFTR* variant remain to be addressed. The microbiota is now included among the additional factors that could contribute to the clinical presentation, pulmonary exacerbations, and heterogeneity of patient responses to treatments. 

Culture-independent profiling of CF airways revealed that (i) the CF respiratory microbiome is a complex community dominated by a more diverse core group of taxa than previously thought [2], and that (ii) stability and resilience are overarching features of the highly variable CF microbiota [3,4]. The CF microbiota includes *Achromobacter*, *Burkholderia*, *Pseudomonas*, *Staphylococcus*, *Stenotrophomonas*, and *Streptococcus* spp. alongside of obligate anaerobes such as *Prevotella* and *Veillonella*, and alongside an increased prevalence of non-tuberculous mycobacteria (NTM), viruses, fungi of the *Aspergillus*, *Candida* or *Malassezia* genera and archaeal phyla in relatively smaller percentages [5]. Overall, even if every individual CF patient harbours a unique and evolving microbial community, an emerging ecological pattern in CF highlights the relationship between decreasing microbiota diversity and worsening lung function [2,6]. Interactions between members of the microbial community cause CF-related lung disease to progress, dictate the response to therapy, and affect clinical outcomes [7,8]. In concert, environmental factors such as diet and lifestyle as well as host genetics define the chemical and physical landscape inhabited by the microbiome, and shape its composition [9,10]. For a comprehensive overview of the current understanding of the structure, composition, and dynamics of CF microbiota, readers are referred to several recent reviews [7,11,12,13].

Conditions that could lead to dysbiosis also exist in the gut of CF patients, where reduced bicarbonate secretion from the pancreas, intestines, and biliary tree are linked to the primary CFTR defect. Regular use of antibiotics due to recurrent pulmonary infections, increased amounts of malabsorbed luminal content, and impaired innate immunity may further contribute to the development of microbial dysbiosis [14]. A significant reduction in gut microbial richness and diversity in samples from CF patients was reported in a number of studies [14,15,16,17,18]. Specifically, the gut microbial communities within the CF population are characterised by reduced abundances of specific bacterial taxa including members of the genera *Clostridium*, *Eubacterium*, *Feacalibacterium*, and *Bacteroides*, as well as the order *Lactobacilliales*. Emerging evidence associates this dysbiosis within the CF gut with seeding of opportunistic pathogens such as potentially pathogenic *Escherichia coli* and *Eubacterium biforme* [19]. In addition, reduced microbial diversity correlates with a shorter time to CF exacerbation and respiratory colonisation by *Pseudomonas aeruginosa* [20]. Some reports have investigated the links between inflammation and bacterial dysbiosis [15,21], but the mechanisms linking microbiome composition to gastrointestinal manifestations of CF remain largely unexplored, and would merit further research [22,23]. 

The CF microbiome has mainly been studied using nucleic acid sequencing technologies, particularly based on sequencing of specific marker amplicons (e.g., 16S rRNA bacterial gene). Due to the lack of universal gene markers for fungi, archaea, and viruses, use of these techniques has led the overwhelming majority of investigations of CF microbiota to focus on bacteria. Recently, shotgun metagenomics emerged in this arena and revealed a great number of insights into other components of the microbiota, thus advancing our knowledge of its composition. Nevertheless, to obtain further insights into microbial function and the interactions between the various constituents, complementary approaches should be applied [24,25].

In this mini-review, taking into account the state-of-the-art CF microbiota research and constant advances in proteomics strategies, we explore how and to what extent proteomics-based approaches could contribute to advancing our knowledge of both respiratory and intestinal microbiota in a CF context, and of host–microbiota crosstalk.

## 2. Can the Current Understanding of the CF Microbiota Shed Light on Genotype–Phenotype Associations?

Microbiomes contribute substantially to the biology of their host and often expand the physiological capacity of the partner organism. In certain cases, these connections have an experimentally identified biochemical basis, whereas in others, these relationships are poorly defined [26,27]. The human microbiome is particularly large and displays significant diversity and complexity, but the lack of appropriate biological models has limited the possibilities for experimentation. 

In the context of CF, a link between *CFTR* gene mutations and modifications to the microbiota was first demonstrated by Cox et al. [28]. These authors reported specific *CFTR* mutations, known to influence the airway environment, and found them to be strongly associated with distinct pathogen profiles. These profiles could potentially explain the varying degrees of severity of pulmonary symptoms commonly observed with various *CFTR* genotypes. Similarly, a link was established also between *CFTR* variants and alterations to faecal microbiota [19], opening the way to studies that perceive CF as a systemic disease, and linking the lung and the gut in a single axis. Despite these pioneering works, both the causes and consequences of dysbiosis for diseases like CF remain difficult to determine. A large number of confounding variables such as antibiotic use, diet, and underlying genetic or environmental factors may significantly contribute to the creation of a distinct niche, and influence the resident microbial community. To this end, the relationships between CFTR dysfunction, microbes, and intestinal health were recently tested in a germ-free CF mouse model [29]. Not only were *CFTR* gene mutations sufficient to alter the microbiome in the gastrointestinal (GI) tract, but they also modulated the host’s adaptive immune response. Concomitantly, host gene–microbiome interactions were identified in the colon of CF patients [30]. Despite this intensive research focus, the complexity of the issue means many questions remain open. For example, how are CF host genetics, microbiome, and host phenotypes linked? What are the relative contributions of the host genome and microbiome to the CF disease phenotype? The highly integrated host physiologies and their associated microbes have increased the popularity of the concept of the holobiont, the consortium consisting of the host plus its associated microbes evolving to function as an inextricable single unit [31]. In CF, genotype–phenotype associations derived from the analysis of both human genetics and the human microbiome are not yet available, and this type of data is rare even in the context of other diseases [32].

## 3. Meta-Omics in CF Microbiota Research

The PubMed database was searched for articles published between January 2007 and 2021 using the terms “metagenomics”, “metatranscriptomics”, “metaproteomics”, “metabolomics” and “cystic fibrosis”. The results revealed that in recent years, several omics techniques were employed to answer a wealth of broad questions linked to CF (Table 1). The majority of these studies focused on determining the components of the CF respiratory microbiome. Only recently, investigations have also begun to explore the CF microbiome in other organs, including the GI tract. As gut dysfunction is another prominent feature of CF, and a growing body of evidence supports the existence of a gut–lung axis [33], findings from these studies may have important clinical implications. Strikingly, transcriptomics and metaproteomics studies have rarely been applied in this field, due to the intrinsic difficulty of the functionally oriented approaches.

### 3.1. Metagenomics Profiling of CF Microbiota 

To date, the majority of culture-independent CF microbiome studies have used 16S ribosomal RNA profiling (metabarcoding targeting prokaryotes), which provides information on the bacteria and some archaea present in a given sample. The speed of analysis and cost effectiveness of this approach mean it is compatible with the analysis of large numbers of samples. However, it has a limited taxonomic resolution and is subject to biases associated with the DNA extraction and PCR steps required to amplify the marker genes [36]. 

**Figure 1:**
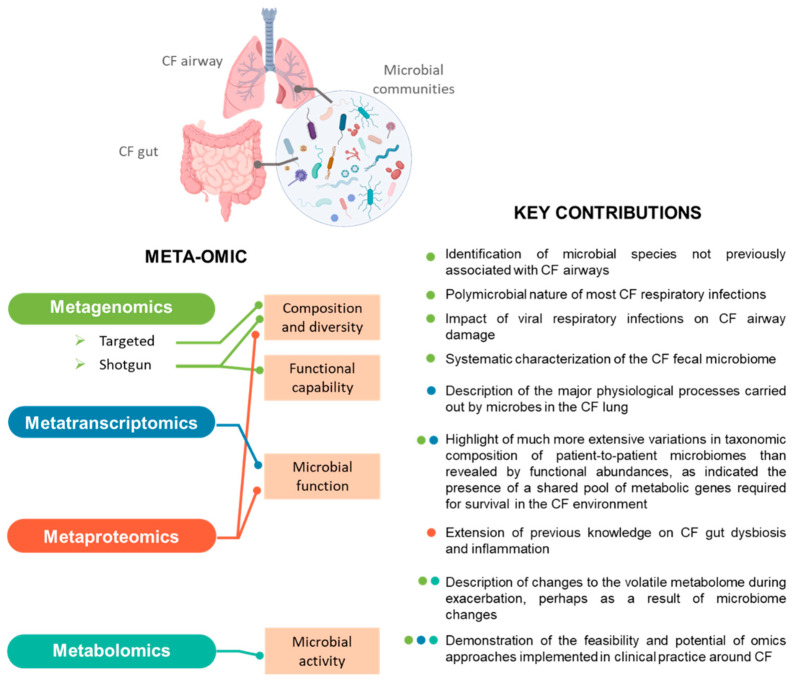
Meta-omics profiling of CF microbiota. For each “omics” technology employed to characterise the respiratory or intestinal microbiota associated with CF, the aspects investigated and key contributions to the field are indicated. The colour of the dots next to each finding listed indicates the technique by which it was obtained.

Untargeted sequencing of DNA extracted from microbial communities, or shotgun metagenomics, enhances the taxonomic and functional resolution of amplicon sequencing and has been shown to surpass the 16S rRNA-based approach when characterising a microbial community [25,37]. Within the CF lung habitat, metagenomics studies reveal a broad repertoire of viruses, moulds, fungi, archaea, and bacteria [38,39,40]. These data allowed the classification of a larger proportion of microbiome inhabitants at the species level and prominent CF pathogens at the strain level, as well as the detection of multiple subpopulations of specific pathogens [39,41,42,43] (Figure 1). Recently, Bacci et al. [42] and Dmitrijeva et al. [40] used shotgun metagenomics approaches to track the temporal dynamics of the sputum microbiome. Their results open up the possibility that such methods could be used to help monitor disease progression by providing routine high-resolution characterisation of the CF lung microbiome. Shotgun metagenomic analysis was also used to systematically characterise the CF faecal microbiome [21], and revealed marked CF-associated taxonomic dysbiosis and functional imbalance. Despite these promising results, the method may be significantly affected by biases in the metagenomic output, similar to those observed during characterisation of the CF respiratory tract biome, because of the presence of a high proportion of human DNA [25,37]. To overcome the attendant limitations and improve metagenomics results, a culture-enriched metagenomic sequencing strategy was investigated by Whelan et al. [44]. Culture enrichment allowed the identification of 63% more operational taxonomic units than direct sequencing, proving that culture-enriched molecular profiling is a valuable strategy for the in-depth characterisation of CF microbiota.

### 3.2. Metatranscriptomics Profiling of CF Microbiota

Metatranscriptomics reveals the transcripts present within a microbiome at a given point, and thus in time provides a snapshot of its active members. Although the implementation of next-generation sequencing (NGS) technologies to RNA significantly increased the scope for applications of this technique [45], applications to CF microbial communities remain limited to less than a handful of reports (Table 1, Figure 1). 

Metatranscriptomics applied to fresh CF sputum revealed similar metabolic profiles between patients highlighting the existence of a shared pool of metabolic genes required for survival in the CF environment [37]. The much more extensive interpatient variation observed in terms of taxonomic composition of the microbiome as compared to functional abundances was corroborated by the comprehensive view of the major physiological processes activated by microbes in the CF lung [46]. Although the technique is biased towards organisms with higher transcriptional rates, the metatranscriptomic profile of the CF microbiome may inform the course of action for treatment and help guide the development of novel therapies through manipulation of the CF environment. This potential for exploitation of metaproteomics data was also illustrated when it was combined with other omics approaches to determine which members of the microbial communities were responsible for a fatal exacerbation [47]. 

However, several challenges remain to be overcome when attempt to apply metatranscriptomics to host-associated microbes. For instance, messenger RNAs (mRNAs) account for only ~5% of total cellular RNA. While various rRNA depletion methods have been developed to enrich the mRNA content of samples, some are not suitable for use with samples containing large amounts of eukaryotic mRNA, such as the CF sputum [37]. In addition, the lack of standardised methods for RNA preparation and storage, or even data analysis further limits the application of the technique when profiling the CF microbiome. 

### 3.3. Metabolomics Profiling of CF Microbiota 

Metabolomics involves the study of metabolites in a biological sample at a given time. Although it remains difficult to determine whether the molecules are of human or microbial origin, a few studies applying metabolomics to CF microbiota have been published (Table 1, Figure 1). Parallel breath metabolomics and shotgun metagenomics revealed that 2,3-butanedione, a fermentation product potentially produced by *Streptococcus* species, the concentration of which varies with antibiotic therapy, is abundant in CF patients and can serve as a cue to neighbouring microbes [48]. These findings, in combination with volatile metabolite profiles associated with common CF pathogens and the host’s immune response to infection, suggest that changes to the volatile metabolome during an exacerbation correlate with changes to the microbiome [49]. Similarly, a study combining metagenomics with two types of untargeted metabolomics analyses applied to sputum samples indicated that the temporary and moderate changes to the lung microbiome observed after initiating the CFTR modulating treatment Ivacaftor/Lumacaftor are mainly characterised by a reduction in the relative abundance of *P. aeruginosa* [50]. A branch of metabolomics dedicated to lipid analysis [51] (lipidomics) studies the lipids present in a sample, arising from host cells and tissues/lipid substrates [52]. Interestingly, when this technique was applied to the CF airway, it raised the question as to the possible involvement of some components of the microbiota in the synthesis and metabolism of some of the lipids identified. This data fuelled what remains a fertile area of investigation [52]. 

With increasing evidence showing a link between gastrointestinal microbiota and the progression of lung disease in CF [53,54], metabolomics was also employed to investigate the association between an altered functional environment and gut dysbiosis [55,56]. Although recent studies have shown that supplementation with probiotics like *Lactobacillus* could decrease pulmonary exacerbations [15,57], the temporal changes and clinical significance of gut dysbiosis and its effects on the disease process remain poorly understood [54]. In this context, targeted investigation and quantification of metabolites could improve our understanding of how the respective metabolic activity of the lung and gut microbiota affect respiratory health [58]. Furthermore, by establishing distinct microbiome and metabolome signatures, a more personalised approach to patient diagnosis and treatment can be proposed [13,47]. 

## 4. (Meta) Proteomics-Based Approaches Applied to CF Microbiota: Where Are We Today?

### 4.1. Metaproteomics Profiling of CF Microbiota 

Proteomics is a multi-faceted omics approach because of the myriad of options it provides to study proteins from various perspectives: abundance, post-translational modifications, quaternary structure, interactions, subcellular localisation, tissue-specific expression, and half-life. Discovery proteomics has extensively contributed to CF research, from the understanding of CFTR biology [59] to the discovery of biomarker for CF diagnosis [60,61,62]. Proteomics-based CF research has used a wide range of techniques including two-dimensional gel electrophoresis (2-DE), liquid chromatography, mass spectrometry (MS), and antibody/protein microarrays. These techniques have been applied to analyse secretions, cells, and whole tissues from in vitro or in vivo disease models, human subjects, and infecting microorganisms, as well as, to a lesser extent, to profile the CF microbiota. Recent progress in MS has made the identification and quantification of thousands of proteins possible in a single shotgun proteomics study. In this approach, the proteins are proteolysed with trypsin and the resulting peptides are resolved by reverse phase chromatography before their identification by tandem mass spectrometry (MS/MS) [63,64]. This approach is compatible with differential comparative studies and can be used to identifying the key players in various conditions.

Over the past decade, metaproteomics [64], defined as the large-scale profiling of the whole protein complement produced by a complex microbial ecosystem, has been used to analyse human microbiota from distinct anatomical sites. These type of analyses are gradually gaining momentum as they unravel the functionality of the complex microbial consortium in situ. Metagenomics provides insights into the taxonomic composition, but is restricted to a prediction of the functional potential of the samples analysed. In contrast, metaproteomics can reveal the actual functional profile and provide an in-depth view of the interplay between microorganisms and their host or environment [65,66]. Moreover, having provided information on the role of microbiota in healthy individuals, the field has progressed to explore the functional profiles of disease-related dysbiosis [67]. These data contribute to our understanding of the underlying pathophysiology and pave the way for targeted approaches to improve health. 

Notwithstanding its potential, to date, proteomics-based CF microbiota profiling is limited to the work of Debyser et al. [34,35] (Table 1, Figure 1). Applying LC-MS to comparatively analyse stool protein extracts from CF patients and their healthy siblings as controls, these authors extended previous knowledge on gut dysbiosis and inflammation observed in CF patients. Thus, they reported CF to be associated with lower numbers of butyrate-producing bacteria and increased numbers of *Enterobacteriaceae*, *Ruminococcus gnavus*, and some clostridia. These species could therefore be viewed as markers of intestinal dysbiosis in patients with CF. In addition to these elements, their metaproteomics approach revealed a list of host proteins associated with inflammation and mucus production that, in combination with bacterial proteins, could serve as LC-MS biomarkers. These biomarkers could be used to monitor gut health in patients with CF, particularly to track responses to probiotic or chemotherapeutic interventions. 

Although it has not yet been applied to characterise respiratory microbiota in CF patients, the metaproteomics approach has been used in the context of other respiratory diseases. These studies demonstrated that it was possible to identify microbial peptides, bacterial taxa, and associated biological functions activated by the microbial community in the distal lung [68]. Recently, Pathak and colleagues [69] used metaproteomics to identify changes in pathogen and host gene expression in patients with ventilator-associated pneumonia (VAP). They identified 62 unique pathogen-produced peptides in VAP patients compared to controls and more than 3000 human proteins, many of which are associated with innate and adaptive immunity. These findings provide mechanistic insights into host–pathogen interactions associated with VAP and could therefore contribute to facilitating diagnosis and treatment. 

### 4.2. Methodological Considerations When Profiling CF Microbiota by Metaproteomics: Data Acquisition

Proteomics data are typically generated by MS following a workflow such as that reviewed by Lin et al. [70]. In metaproteomics, proteins are commonly measured using a bottom-up approach involving extraction, isolation, and digestion of proteins to produce peptides. These peptides are then separated and analysed using liquid chromatography coupled to tandem mass spectrometry (LC-MS/MS). The resulting MS/MS spectra are typically matched against in silico generated spectra derived from a protein sequence database to identify the peptides detected and infer the identities of the original proteins. The inferred proteins are then used to determine which taxa are active in the community, their functions, and the relative gene expression levels. Each of the steps in this process can potentially influence the outcome of a metaproteomics analysis, as well as providing its own specific benefits and challenges [71]. Overall, two major challenges still limit the routine application of metaproteomics: (i) sample preparation due to high sample complexity and extensive contamination, and (ii) data analysis due to the computational efforts required to treat large datasets, a lack of appropriate annotated protein sequence databases, and difficulties with protein inference leading to ambiguous protein annotation [72].

**Figure 2:**
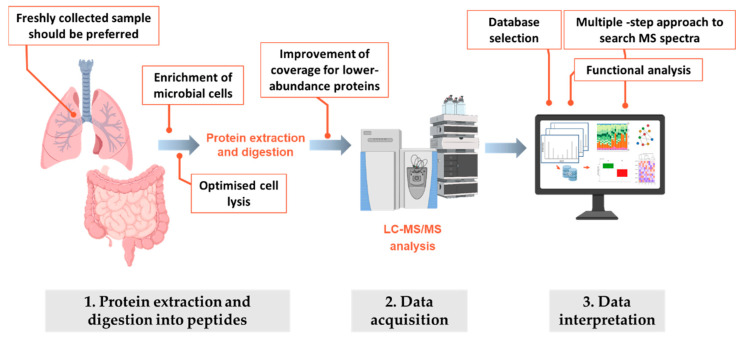
Schematic workflow employed in metaproteomics experiments. For each one of the three main steps in the metaproteomic analysis, critical aspects that should be taken into account or carefully evaluated are highlighted.

Among the factors hindering the use of metaproteomics to profile the CF microbiota, particularly the respiratory one, we can include the nature of the CF samples. Compared to the microbiota inhabiting other anatomical sites, the respiratory microbiota is a low biomass community, extensively contaminated with proteins from epithelial cells or cells associated with the acute immune response triggered by diseases such as CF. Because of the high percentage of host proteins, sample pre-treatment steps must be applied to increase the recovery of microbial relative to human biomass. Several methods to enrich and purify microbiota cells, and their potential biases, were recently reviewed [73]. Based on this review, freshly collected samples should be preferred to avoid uncontrolled microbial lysis caused by freezing and thawing of samples. This lysis potentially negatively affects recovery of the proteins of interest, as does the complexity of the very thick and sticky CF mucus [74], and the use of antibiotics [75]. In addition, as suggested by Jagtap et al. [68], coverage of lower-abundance proteins should be improved by depleting known medium- and high-abundance proteins (Figure 2). Similarly, the need for a step to enrich microbial proteins to allow comprehensive proteomic analysis of faecal samples from CF patients was recently addressed through the development and comparison of two shotgun metaproteomic workflows [34]. The results of this comparison revealed the huge influence of the protein extraction method used on the results obtained, underlying the need for a standardised and tailored sample preparation for metaproteomics analysis.

### 4.3. Methodological Considerations When Profiling CF Microbiota by Metaproteomics: Data Interpretation

The analysis of metaproteomics datasets presents several computational challenges that must be dealt with to ensure reliable peptide and protein identification and support the interpretation of results with meaningful protein annotations. The unique challenges and pitfalls facing researchers at each step of the analysis have been reviewed by several authors. Among others, Starr et al. [73], Heyer et al. [72], and more recently Schiebenhoefer et al. [76] provide comprehensive, detailed, and flexible guides to analysing metaproteomics data. Despite the challenges outlined, the added value of metaproteomics compared to other omics approaches has already been demonstrated numerous times. Thus, in the environmental and medical fields, it has provided unprecedented insights into the functional activity of microbial communities [64]. Recent advances in interpretation of metaproteomics data, such as trough application of the phylopeptidomics concept to allow precise estimation of the biomass contribution of each microorganisms identified in a given sample, add additional value to what can be obtained from each sample [77]. Although complementary to the more commonly used nucleic acid sequencing-based methods, the information provided by metaproteomics inherently differs from the approximations of cell or genome copy counts [66]. Furthermore, culturomics and proteotyping, a proteomics-derived methodology for rapid identification of microbial isolates, could provide a wealth of novel information on the CF microflora [78]. Because of the direct link between phenotypic traits and proteome characteristics, insights into the physiological state and functions of microbial populations could be obtained by proteotyping microorganisms grown under differing culture conditions. 

## 5. Perspectives for Applications of Proteomics-Based Approaches to CF Microbiota

**Figure 3:**
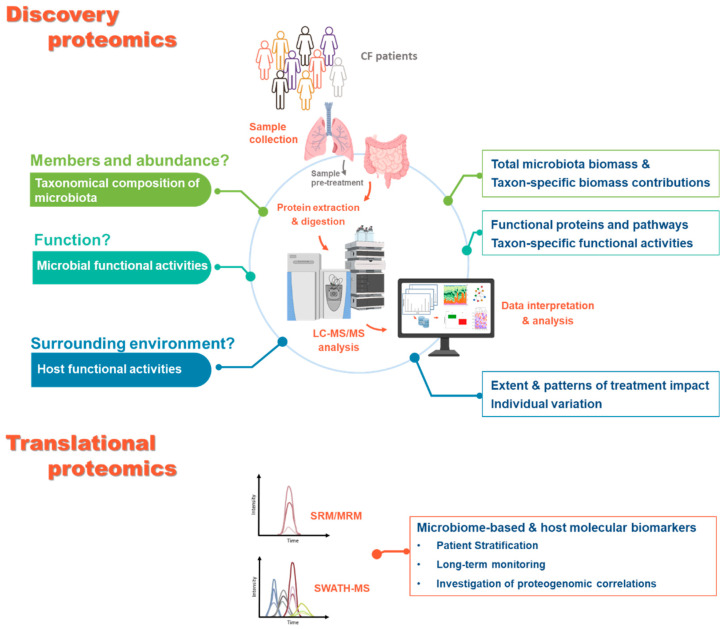
Questions that could be answered using proteomics-based approaches. Sample questions that could currently be addressed by analysing airways and intestinal microbiota from CF patients using proteomics-based approaches. For the discovery proteomics, for each question described (**left** side), the corresponding output of the metaproteomic analysis is summarised (**right** side). The main steps in the experimental workflow and the widely used approaches are also highlighted. For the translational proteomics, the main approaches are indicated alongside with their output.

How can proteomics improve our understanding of CF phenotypic variability and more generally of CF disease? Figure 3 shows the set of questions that we believe can now by developing new aspects such as relative biomass estimation and improved functional integration. Like for other microbiota, the contribution of both the airway and the faecal microbiota to CF can be considered to be only partially explored when the taxonomy of their members is considered in isolation. Today, the degree of functional diversity of the microbiota associated with extensive interpatient variability in CF remains underexplored, and inferences between changes to microbial composition and disease phenotypes have been based on associations and correlations, with little attempt to explain the underlying mechanisms [79]. In this scenario, metaproteomics profiling of the CF microbiota (discovery proteomics), in addition to allowing analysis of the composition of the microbiota by quantifying the biomass contributions of individual species, could provide a parallel assessment of the specific functional roles of microbes in a defined microbial community. It thus has the potential to reveal their relevance to the clinical course of CF (Figure 3).

In parallel to characterisation of the microbiota, thanks to the analysis of proteins of all origins, metaproteomics provides insights into the molecular functions attributed to host proteins. These functions can then infer the host–microbiome interactions underlying the disease. Metaproteomics assessment of CF samples also holds great promise for the integration of microbiota information as part of CF infection management. Host proteins could inform on changes to the inflammatory state, the host immune response, and more generally on how therapies affect disease trajectory. Inflammation of the airways is a common biological response to damage or infection and, though generally beneficial, it can significantly influence the composition of the airway microbiome [80]. In CF airways, increased secretion of proinflammatory mediators (e.g., interleukin (IL)-6 and IL-8) by potentially defective airway epithelium and immune cells causes a disproportionate influx of neutrophils. The subsequent release of proteases, such as neutrophil elastase, can damage structural lung proteins, leading to an inevitable decline in pulmonary function. Thus, the development of a range of microbiome-based molecular biomarkers could be useful in monitoring the onset or progression of airway disease, giving clinicians advance warning of changing conditions, and guiding their therapeutic interventions [13] (Figure 3). With therapies that correct the underlying defects in CFTR proteins either available or in the pipeline, there has been great interest in profiling their effect on microbiota. In this context, proteomics approaches alone and in association with metabolomics could provide insights into the correlation between taxonomic and functional dynamics in the microbiota and the efficacy of CFTR modulating therapies. In addition, it could identify potential biomarkers (microbes and/or key proteins) to predict the efficacy of a treatment and/or serve as new therapeutic targets. 

In addition to expanding our understanding of CF microbial communities, proteomics, particularly its subdiscipline “targeted proteomics”, supports the translation to clinical application of candidate biomarkers identified during the discovery phase. Currently, MS techniques such as SRM/MRM (selected reaction monitoring or multiple reaction monitoring) and SWATH (sequential window acquisition of all theoretical mass spectra) can be used to quantify proteins present in biofluids, tissues, cells, and faeces. This quantification can contribute to biomarker discovery and validation, patient stratification, monitoring of clinical trials, and investigation of proteogenomic correlations (Figure 3). Although they have not yet been applied in the context of CF, the successful use of translational proteomics methods in several proteomics and microbiome studies [70,71,73] underlines their considerable potential.

The recent development of proteomics and metaproteomics technologies should provide additional valuable insights into how microbiota contribute to the progression of CF disease, and its interaction with the host. Moving away from taxonomic inventories towards a better understanding of how the CF microbiota functions could open a new avenue for the identification of novel biomarkers of CF progression and targets for antimicrobial therapy or microbiota-based interventions. However, the true value of proteomics approaches is revealed through integration with other -omics technologies, including genomics, epigenomics, lipidomics, and metabolomics. Ultimately, only by combining the knowledge gained in each of these fields will we be able to truly elucidate the complex interplay between the microbiome and its host in CF.

## Figures and Tables

**Table 1:** Survey of CF-related “omics” publications *.

Molecular Level (Methodology)	Biological Questions Addressed	CF Associated Records
DNA (metagenomics)	What is the ecology of the CF lung microbiome and the ecological patterns of CF microbiota?	108
mRNAs (transcriptomics)	Which genes are expressed? Which components of the microbiota are active?	3
Proteins (metaproteomics)	What are the key players in the CF lung? Could such proteins be biomarkers of exacerbation?	2 **
Metabolites (metabolomics)	How does the gut microbiota affect host metabolism?	128
Lipids (lipidomics)	Is there a lipid signature associated with CF progression?	22

* PubMed search results. This is not a systematic review. ** [34,35].

## Data Availability

Not applicable.
